# Impacts of COVID-19 on hemostasis: coagulation abnormalities and management perspectives

**DOI:** 10.1097/MS9.0000000000002237

**Published:** 2024-07-02

**Authors:** Emmanuel Ifeanyi Obeagu, Muhammad Tukur, Kingsley Akaba

**Affiliations:** a Department of Medical Laboratory Science; b Department of Science Education, Faculty of Education, Kampala International University, Kampala, Uganda; c Department of Haematology, University of Calabar, Calabar, Cross-River State, Nigeria

**Keywords:** anticoagulation, coagulopathy, COVID-19, hemostasis, thrombosis

## Abstract

The COVID-19 pandemic caused by SARS-CoV-2 has transcended its initial characterization as a respiratory illness, revealing substantial implications for hemostasis and coagulation pathways. COVID-19-associated coagulopathies have emerged as critical determinants of disease severity and prognosis, presenting a multifaceted challenge in clinical management. This paper aims to elucidate the intricate interplay between COVID-19 and hemostasis, delving into the underlying mechanisms of coagulation abnormalities, exploring the spectrum of thrombotic complications, and discussing evolving management strategies. Therapeutic interventions and anticoagulation strategies tailored for managing COVID-19-related coagulopathies form a significant focus, encompassing prophylactic and therapeutic approaches, heparin-based therapies, and individualized treatment paradigms. This paper underscores the imperative for ongoing research endeavors to refine diagnostic modalities, identify novel therapeutic targets, and ascertain long-term sequelae of COVID-19-induced coagulation abnormalities. Ultimately, a comprehensive understanding of the intricate relationship between COVID-19 and hemostasis is pivotal in devising effective management strategies to mitigate thrombotic risks, improve clinical outcomes, and pave the way for tailored interventions in affected individuals.

## Introduction

Highlights

- Increased risk of thrombosis: COVID-19 has been associated with a higher incidence of thrombotic events.
- Endothelial dysfunction: The virus can directly infect endothelial cells, leading to endothelial dysfunction and activation.
- Hypercoagulability: COVID-19 induces a state of hypercoagulability characterized by elevated levels of fibrinogen, D-dimer, and other coagulation markers.
- Disseminated intravascular coagulation (DIC): Severe cases of COVID-19 may progress to DIC.
- Impact on management strategies: Coagulation abnormalities in COVID-19 patients necessitates careful monitoring and management of anticoagulation therapy.

The COVID-19 pandemic caused by SARS-CoV-2 has evolved into a global health crisis, unraveling a spectrum of clinical manifestations extending beyond respiratory compromise. Notably, COVID-19 has revealed its profound impact on the intricate balance of hemostasis and coagulation, transcending traditional paradigms and significantly affecting disease outcomes^1–6^. Initially characterized by respiratory symptoms, COVID-19 has demonstrated a multifaceted pathogenesis involving diverse systemic effects. One critical facet that has gained prominence is the propensity for coagulation abnormalities and thrombotic complications, leading to severe morbidity and mortality in afflicted individuals. Understanding the complex interplay between SARS-CoV-2 infection and the hemostatic system is crucial in elucidating the underlying mechanisms, prognostic implications, and evolving therapeutic strategies^7–11^.

This paper aims to explore the multifaceted impacts of COVID-19 on hemostasis, specifically focusing on coagulation disturbances, thrombotic risks, and evolving management perspectives. We embark on this journey by dissecting the intricate pathophysiological mechanisms underlying COVID-19-associated coagulopathies, including the role of endothelial dysfunction, inflammatory responses, and viral interactions in perturbing the delicate balance of hemostatic regulation. A critical examination of the diverse thrombotic manifestations observed in COVID-19, ranging from venous thromboembolism and disseminated intravascular coagulation to microvascular thrombosis and arterial thrombotic events, follows suit. This exploration illuminates the clinical ramifications of these coagulation disturbances, unraveling their profound implications for disease severity, prognosis, and therapeutic interventions.

Ultimately, this paper aims to underscore the imperative for ongoing research endeavors to refine diagnostic modalities, identify novel therapeutic targets, and elucidate the long-term consequences of COVID-19-induced coagulation disturbances. A comprehensive understanding of the intricate relationship between COVID-19 and hemostasis is paramount in devising effective management strategies, thereby mitigating thrombotic risks, improving clinical outcomes, and guiding tailored interventions in affected individuals.

## Hemostatic alterations in COVID-19

COVID-19, caused by the novel coronavirus SARS-CoV-2, has presented an array of systemic manifestations, prominently involving hemostasis and the coagulation cascade. Emerging evidence underscores the multifaceted disruptions in hemostatic balance observed in affected individuals, encompassing a spectrum of coagulation abnormalities and thrombotic complications^12–15^. One of the pivotal pathophysiological mechanisms underlying COVID-19-related coagulopathy involves endothelial dysfunction^16^. SARS-CoV-2’s affinity for the angiotensin-converting enzyme 2 (ACE2) receptors expressed on endothelial cells triggers a cascade of events, inciting endothelial injury and perturbing vascular homeostasis. Endothelial dysfunction leads to dysregulated thromboinflammatory responses, characterized by increased von Willebrand factor release, impaired fibrinolysis, and activation of prothrombotic pathways, culminating in a hypercoagulable state^17^. Table 1 shows hemostatic alterations in COVID-19.

**Table 1:** Hemostatic alterations in COVID-19.

Hemostatic alteration	Description
Hypercoagulability	Increased risk of blood clot formation, leading to conditions like deep vein thrombosis (DVT) and pulmonary embolism (PE)
Disseminated intravascular coagulation (DIC)	Severe cases may develop DIC, a condition where the normal blood clotting process becomes overactive, leading to widespread clot formation and consumption of clotting factors
Elevated D-dimer levels	D-dimer is a marker of fibrinolysis and elevated levels indicate increased breakdown of blood clots. Elevated D-dimer is commonly seen in severe COVID-19 cases
Thrombocytopenia	Reduction in platelet count, which can contribute to bleeding tendencies and other complications
Endothelial dysfunction	The virus can damage the endothelial cells lining blood vessels, leading to impaired regulation of clotting and fibrinolysis
Altered levels of clotting factors	Changes in the levels of various clotting factors, such as increased von Willebrand factor and decreased antithrombin III, contributing to the hypercoagulable state
Hypercoagulability	Increased risk of blood clot formation, leading to conditions like deep vein thrombosis (DVT) and pulmonary embolism (PE)

The dysregulated immune response in severe COVID-19 is marked by an inflammatory cascade involving elevated proinflammatory cytokines, often referred to as a “cytokine storm.” This exaggerated cytokine release, including interleukin-6 (IL-6), tumor necrosis factor-alpha (TNF-alpha), and others, contributes to systemic inflammation and triggers coagulation cascades, promoting a prothrombotic milieu. Additionally, the imbalance between procoagulant and anticoagulant factors, such as increased tissue factor expression and reduced antithrombin levels, exacerbates the hypercoagulable state observed in severe cases^18–22^.

Direct interactions between SARS-CoV-2 and the coagulation cascade components have been implicated in provoking coagulation abnormalities. The virus appears to activate platelets, inducing platelet aggregation and releasing prothrombotic factors, further exacerbating the procoagulant state^23,24^. COVID-19-induced alterations in hemostatic factors involve disrupted levels of various markers, including increased D-dimer, fibrinogen, and factor VIII, alongside decreased levels of natural anticoagulants like antithrombin and protein C. These imbalances collectively contribute to the prothrombotic phenotype observed in severe COVID-19 cases^25,26^.

## Coagulation abnormalities and thrombotic complications

COVID-19 has been associated with a spectrum of coagulation abnormalities that significantly impact disease severity and clinical outcomes. The dysregulated coagulation cascade in severe cases of COVID-19 manifests as a prothrombotic state, leading to diverse thrombotic complications across multiple vascular beds. Venous thromboembolism, including deep vein thrombosis (DVT) and pulmonary embolism (PE), represents a critical thrombotic manifestation observed in COVID-19 patients. Severe endothelial dysfunction, heightened coagulation activation, and immobilization-related factors contribute to an increased incidence of VTE, particularly in critically ill individuals^27,28^. DIC, characterized by systemic activation of coagulation pathways leading to both bleeding and thrombotic tendencies, has been reported in severe COVID-19 cases. The complex interplay between systemic inflammation, endothelial injury, and coagulation dysregulation contributes to the pathogenesis of DIC in this setting^20–29^.

COVID-19-associated coagulopathy involves microvascular thrombosis, characterized by the formation of microthrombi in small blood vessels. These microthrombi contribute to tissue ischemia, and organ dysfunction, and may explain the heterogeneous clinical manifestations observed in affected individuals^14,30^. While venous thrombotic events predominate, arterial thromboses, including strokes, myocardial infarctions, and limb ischemia, have also been reported in COVID-19 patients. Enhanced platelet activation, endothelial dysfunction, and prothrombotic factors collectively contribute to the increased risk of arterial thrombotic events. Thrombotic complications in COVID-19 significantly impact disease severity, prognosis, and mortality rates. Patients presenting with thrombotic events often exhibit more severe disease phenotypes, highlighting the prognostic implications of coagulation abnormalities in COVID-19^29^. The heightened thrombotic risks observed in COVID-19 necessitate vigilance in thromboprophylaxis and anticoagulation strategies. Tailored approaches considering individual risk factors, disease severity, and balancing thrombotic risks against bleeding complications are imperative in optimizing clinical outcomes^30^.

## Mechanisms of endothelial dysfunction

COVID-19 pathogenesis involves intricate interactions between SARS-CoV-2 and the endothelium, leading to widespread endothelial dysfunction. Endothelial cells, lining the vasculature, play a pivotal role in maintaining vascular homeostasis, regulating hemostasis, and modulating inflammatory responses. In severe COVID-19 cases, viral invasion and the ensuing inflammatory milieu trigger several mechanisms contributing to endothelial dysfunction^31–33^. SARS-CoV-2 gains entry into host cells through binding to angiotensin-converting enzyme 2 (ACE2) receptors, abundantly expressed on endothelial cells. Viral invasion induces direct endothelial injury, disrupting endothelial cell integrity and function, consequently compromising vascular homeostasis^34,35^.

The dysregulated immune response characteristic of severe COVID-19 involves a cytokine storm, marked by an excessive release of proinflammatory cytokines (e.g. IL-6,TNF-alpha). These cytokines elicit endothelial activation, upregulating adhesion molecules, such as selectins and integrins, promoting leukocyte recruitment and endothelial cell adhesion, contributing to endothelial dysfunction^36^. COVID-19-related endothelins, characterized by endothelial inflammation and cellular injury, disrupt the endothelial barrier integrity. This endothelial dysfunction leads to increased permeability, impaired vasoregulation, and altered hemostasis, contributing to a prothrombotic phenotype^16^. The disrupted endothelial balance between vasodilatory and vasoconstrictive factors, including nitric oxide (NO), endothelin-1, and prostacyclin, contributes to vasoconstriction, impaired vascular tone regulation, and microvascular dysfunction, further exacerbating endothelial injury. Endothelial dysfunction in COVID-19 induces a prothrombotic endothelial state, characterized by increased expression of tissue factor, von Willebrand factor, and impaired thrombomodulin and antithrombin activity, promoting coagulation activation and thrombus formation^37^.

## Diagnostic challenges and biomarkers

Accurate and timely diagnosis of COVID-19-related coagulopathies presents significant challenges due to the complex and multifactorial nature of the disease. Several diagnostic dilemmas and the search for reliable biomarkers persist in the identification and stratification of coagulation abnormalities in affected individuals: Elevated D-dimer levels serve as a frequently observed biomarker in COVID-19, indicating ongoing fibrinolysis and potential thrombotic events. However, while elevated D-dimer levels correlate with disease severity, their specificity in distinguishing between thrombotic and non-thrombotic causes remains a challenge^38^. Routine coagulation tests, including prothrombin time (PT), activated partial thromboplastin time (aPTT), and platelet counts, offer insights into coagulation status. However, interpretation challenges arise due to alterations in these parameters influenced by factors such as inflammation, hemodilution, and therapeutic interventions, limiting their specificity for diagnosing coagulopathies in COVID-19.

Markers associated with endothelial dysfunction, such as von Willebrand factor antigen, soluble P-selectin, and thrombomodulin, hold promise as indicators of endothelial injury and coagulation abnormalities in COVID-19. These biomarkers reflect endothelial activation and may assist in prognostication and risk stratification^39^. Ongoing research endeavors are exploring novel biomarkers and molecular profiles that hold the potential to elucidate the pathophysiology of COVID-19-associated coagulopathies. Biomarkers such as soluble urokinase plasminogen activator receptor (suPAR), markers of neutrophil activation, and cytokine profiles may provide insights into disease severity and coagulation dysregulation. Radiological imaging techniques, including computed tomography pulmonary angiography (CTPA), and ultrasound, play a crucial role in detecting thrombotic events, particularly pulmonary embolism and deep vein thrombosis. However, challenges exist in routine implementation due to resource constraints and the risk of nosocomial transmission.

## Anticoagulation strategies and management

The hypercoagulable state observed in severe COVID-19 cases has prompted the exploration and refinement of anticoagulation strategies, aiming to mitigate thrombotic risks while balancing the potential for bleeding complications. Tailored anticoagulation approaches have emerged as crucial components in managing coagulopathies associated with COVID-19: Prophylactic anticoagulation with low molecular weight heparin (LMWH) or unfractionated heparin (UFH) is recommended in hospitalized COVID-19 patients, particularly those at increased risk of thromboembolic events^40^. Risk assessment tools, such as the D-dimer levels and clinical scoring systems, aid in stratifying patients who would benefit from prophylactic anticoagulation.

In patients with severe COVID-19 and high thrombotic risk, intermediate or therapeutic anticoagulation dosing regimens have been considered. However, the decision to escalate anticoagulation to therapeutic levels warrants careful consideration, balancing the potential benefits against the risk of bleeding complications^41^. Risk stratification based on individual patient characteristics, disease severity, comorbidities, and biomarker profiles plays a pivotal role in tailoring anticoagulation strategies. Personalized approaches aim to optimize thromboprophylaxis while minimizing bleeding risks, necessitating ongoing clinical assessment and adjustments in anticoagulant dosing^42^. DOACs, such as apixaban, rivaroxaban, and dabigatran, have shown promise in specific clinical scenarios. Their oral administration and predictable pharmacokinetics offer potential advantages; however, clinical evidence supporting their use in COVID-19-associated coagulopathies remains evolving^43^. Regular monitoring of coagulation parameters, including D-dimer levels and platelet counts, alongside clinical assessments, aids in evaluating response to anticoagulation and identifying potential adverse events, facilitating timely intervention in case of bleeding complications.

## Individualized treatment and risk stratification

Managing coagulopathies in COVID-19 necessitates a nuanced and individualized approach, integrating risk stratification strategies and personalized treatment paradigms tailored to the patient’s clinical profile, disease severity, and thrombotic risks: A comprehensive clinical assessment encompassing patient demographics, comorbidities, disease severity, laboratory parameters (such as D-dimer levels), and imaging findings guides risk stratification and informs individualized treatment decisions. Risk stratification tools aid in identifying individuals at heightened thrombotic risk, facilitating targeted interventions. Elevated D-dimer levels, severe disease presentation, older age, obesity, and comorbidities such as cardiovascular disease or cancer serve as key risk factors warranting vigilant monitoring and potential adjustment of anticoagulation strategies^42^.

Individualized anticoagulation strategies involve selecting appropriate dosing regimens (prophylactic, intermediate, or therapeutic) based on the assessed thrombotic risk and bleeding considerations. Balancing the risks and benefits of anticoagulation, particularly in critically ill patients, requires ongoing assessment and potential adjustments in treatment approaches^43^. Incorporating biomarkers reflecting endothelial dysfunction or specific coagulation abnormalities, alongside radiological imaging modalities [such as computed tomography (CT) angiography, ultrasound], aids in risk stratification, thrombotic assessment, and informing treatment decisions.

Regular monitoring of coagulation parameters, clinical status, and adverse events is pivotal in evaluating treatment response and adjusting anticoagulation regimens as needed. Adherence to standardized protocols and close interdisciplinary collaboration among healthcare providers is essential in optimizing patient care^41^. Engaging in shared decision-making involving patients or their proxies regarding the risks and benefits of anticoagulation fosters patient-centered care. Transparent communication and education regarding the rationale behind individualized treatment approaches enhance patient understanding and compliance^44–50^. Table 2 shows management strategies for coagulation abnormalities in COVID-19 Patients, Table 3 shows challenges in managing coagulation abnormalities in COVID-19 Patients, Table 4 shows emerging therapeutic approaches for coagulation abnormalities in COVID-19, Table 5 shows Pathogenesis of COVID-19-associated coagulopathy, Table 6 shows treatment strategies for COVID-19-associated coagulopathy and Table 7 shows Outcomes of COVID-19-associated coagulopathy.

**Table 2:** Management strategies for coagulation abnormalities in COVID-19 patients.

Management strategy	Description
Anticoagulant therapy	Type, dose, duration
Monitoring parameters	D-dimer levels, platelet count, PT/INR
Role of thromboprophylaxis	Pharmacological and mechanical methods
Individualized treatment approach	Tailoring therapy based on risk stratification

PT-prothrombin time, INR- international normalized ratio

**Table 3:** Challenges in managing coagulation abnormalities in COVID-19 patients.

Challenge	Description
Lack of consensus guidelines	Variability in treatment approaches
Bleeding risk vs. thrombosis risk	Balancing anticoagulation
Laboratory resource constraints	Availability of tests and timeliness of results
Patient-specific factors	Comorbidities, renal or hepatic impairment

**Table 4:** Emerging therapeutic approaches for coagulation abnormalities in COVID-19.

Therapeutic approach	Description
Targeting inflammatory pathways	Anti-IL-6, IL-1 antagonists
Novel anticoagulant agents	Direct oral anticoagulants (DOACs), factor Xa inhibitors
Adjunctive therapies	Fibrinolytics, immunomodulators
Role of convalescent plasma	Potential for immunomodulation and hemostasis

IL, interleukin.

**Table 5:** Pathogenesis of COVID-19-associated coagulopathy.

Mechanism	Description
Endothelial dysfunction	SARS-CoV-2 infects endothelial cells via ACE2 receptors, leading to endotheliitis and disruption of vascular integrity
Cytokine storm	Dysregulated immune response results in excessive release of proinflammatory cytokines (e.g. IL-6, TNF-α), contributing to systemic inflammation and coagulation activation
Platelet activation	Viral-induced inflammation and endothelial injury trigger platelet activation, leading to thrombus formation and platelet consumption.
Coagulation cascade dysregulation	Disruption of coagulation homeostasis, with increased levels of D-dimer, fibrinogen, and von Willebrand factor, and decreased levels of antithrombin, predisposing to thrombosis

ACE2, angiotensin-converting enzyme 2; IL, interleukin; TNF-α, tumor necrosis factor-alpha.

**Table 6:** Treatment strategies for COVID-19-associated coagulopathy

Intervention	Description
Anticoagulation	Prophylactic anticoagulation with low molecular weight heparin (LMWH) or unfractionated heparin (UFH) to prevent thrombotic complications
Antiplatelet therapy	Aspirin or other antiplatelet agents may be considered in select patients to mitigate platelet activation and thrombotic risk
Immunomodulatory agents	Anti-inflammatory agents (e.g. corticosteroids, IL-6 inhibitors) to attenuate cytokine storm and reduce systemic inflammation
Endothelial protection	Agents targeting endothelial dysfunction (e.g. ACE inhibitors, statins) to preserve vascular integrity and mitigate thrombotic risk

ACE, angiotensin-converting enzyme; IL, interleukin.

**Table 7:** Outcomes of COVID-19-associated coagulopathy

Outcome	Description
Thrombotic events	Increased risk of venous thromboembolism (VTE), arterial thrombosis, and microvascular thrombosis, contributing to morbidity and mortality
Bleeding complications	Anticoagulant therapy may predispose to bleeding complications, including gastrointestinal bleeding, intracranial hemorrhage, and surgical site bleeding
Mortality	COVID-19 patients with coagulation abnormalities, particularly those with severe disease and multiorgan dysfunction, have higher mortality rates compared to those without
Long-term sequelae	Persistent coagulation abnormalities and thrombotic risk post-recovery, as well as potential for chronic vascular and organ damage

## Importance and value of prolonged prothrombin time in assessing patient severity on admission

The prolonged PT is a significant laboratory finding that can provide valuable insights into the severity of a patient’s condition upon admission, particularly in the context of COVID-19 and other critical illnesses. Prolonged PT indicates dysfunction in the extrinsic and common coagulation pathways, reflecting a state of coagulopathy. In COVID-19 patients, coagulopathy is increasingly recognized as a significant complication associated with disease severity and poor outcomes. COVID-19 patients are at an increased risk of thrombotic events, including deep vein thrombosis (DVT), pulmonary embolism (PE), and disseminated intravascular coagulation (DIC). Prolonged PT serves as an indicator of potential thrombotic risk, guiding clinicians in implementing appropriate thromboprophylaxis and monitoring strategies. Prolonged PT on admission may reflect the severity of systemic inflammation and multiorgan dysfunction in critically ill patients, including those with severe COVID-19. It can serve as an early warning sign of impending deterioration and the need for intensive care management. Elevated PT levels on admission may serve as a prognostic marker for adverse outcomes, prompting timely intervention and close monitoring. Prolonged PT influences therapeutic decisions, particularly regarding anticoagulation and blood product transfusion. It helps clinicians determine the appropriate dosage of anticoagulants and guide transfusion thresholds for fresh frozen plasma and other blood products in patients with coagulopathy. Serial measurements of PT enable clinicians to monitor the response to therapeutic interventions, including anticoagulation and supportive care measures. Improvements or worsening of PT over time can inform adjustments to treatment strategies and help assess the patient’s trajectory^51–53^.

## Future perspectives and research directions

Advancements in identifying reliable biomarkers and refining diagnostic modalities hold promise in enhancing early detection, risk stratification, and prognostication of COVID-19-related coagulopathies^54–58^. Improved specificity and standardization of biomarkers aid in distinguishing thrombotic from non-thrombotic causes and facilitating timely interventions^59–62^. Deeper insights into the intricate pathophysiological mechanisms underpinning endothelial dysfunction in COVID-19 are pivotal. Assessment of the long-term sequelae of COVID-19-related coagulopathies, including post-acute thrombotic events, chronic endothelial dysfunction, and their impact on cardiovascular health, requires comprehensive follow-up studies in recovered patients. Longitudinal studies elucidating the persistence of coagulation abnormalities and their implications on morbidity and mortality are crucial^1,3,63–70^.

Exploration of potential therapeutic avenues targeting endothelial repair mechanisms, anti-inflammatory strategies, and modulation of coagulation pathways holds promise. Novel therapies aiming to restore endothelial homeostasis and mitigate hypercoagulability merit investigation. Collaborative initiatives fostering data sharing, multinational registries, and standardized protocols facilitate a broader understanding of COVID-19-associated coagulopathies. Interdisciplinary collaboration among researchers, clinicians, and healthcare organizations accelerates knowledge dissemination and facilitates evidence-based practice^31,32,34,35,71–74^.

## Recommendations

Implement standardized protocols for the regular monitoring of coagulation parameters, including D-dimer levels, platelet counts, and fibrinogen levels, especially in hospitalized COVID-19 patients. This aids in early detection of coagulation abnormalities and facilitates timely intervention. Adopt a risk-stratified approach to identify high-risk individuals prone to thrombotic events based on clinical severity, comorbidities, biomarkers, and imaging findings. Tailor anticoagulation strategies accordingly, balancing thrombotic risks against bleeding complications. Continuously reassess and refine anticoagulation protocols, considering the evolving evidence and guidelines. Tailor the choice of anticoagulants, dosage, and duration of therapy based on individual patient profiles and disease severity, aiming for a personalized approach.

Foster collaboration among healthcare institutions, researchers, and global organizations to share data, establish multinational registries, and conduct large-scale studies. This collaborative effort accelerates knowledge acquisition, standardizes practices, and advances understanding of COVID-19-associated coagulopathies. Conduct longitudinal studies to elucidate the long-term consequences of COVID-19-related coagulopathies. Follow-up assessments in recovered patients can help understand post-acute thrombotic risks, chronic endothelial dysfunction, and cardiovascular sequelae. Provide comprehensive education and awareness programs for healthcare professionals regarding the diagnosis, management, and implications of coagulation abnormalities in COVID-19. This empowers clinicians to make informed decisions and implement appropriate interventions.

Invest in research aimed at understanding endothelial dysfunction in COVID-19 and exploring therapeutic interventions targeting endothelial repair mechanisms. This may contribute to restoring vascular homeostasis and mitigating hypercoagulability. Continuously update clinical practices and guidelines based on emerging evidence and expert consensus. Healthcare institutions should adapt and implement these guidelines to ensure standardized and evidence-based care for COVID-19 patients. Engage in shared decision-making with patients and their families, ensuring clear communication about the risks and benefits of anticoagulation strategies. Emphasize the importance of adherence to treatment plans and regular follow-ups for optimal outcomes. Maintain vigilance and adaptability in clinical practices, considering the dynamic nature of COVID-19. Regularly review and update strategies in response to emerging evidence and changing disease patterns.

## Conclusion

The COVID-19 caused by SARS-CoV-2 has evolved into a global health crisis, revealing intricate implications for coagulation and hemostasis. COVID-19-associated coagulopathies represent a complex interplay between viral pathogenesis, inflammatory responses, endothelial dysfunction, and dysregulated coagulation cascades, contributing significantly to disease severity and clinical outcomes.

COVID-19-associated coagulopathies pose significant challenges in clinical management, warranting a multifaceted approach integrating risk stratification, personalized treatment paradigms, and ongoing research endeavors. Understanding the intricate interplay between viral pathogenesis and coagulation disturbances is pivotal in devising effective management strategies, ultimately aiming to mitigate thrombotic risks, improve clinical outcomes, and pave the way for tailored interventions in affected individuals.

## Ethical approval

Not applicable as this a review.

## Consent

Not applicable as this a review.

## Source of funding

No funding was received for writing this review paper.

## Author contribution

E.I.O. performed the following roles: conceptualisation, methodology, supervision, draft writing, editing and approval before submission.

## Conflicts of interest disclosure

The author declares no conflict of interest.

## Research registration unique identifying number (UIN)

Not applicable as this a review.

## Guarantor

Not applicable as this a review. It does not have any data.

## Data availability statement

Not applicable as this a review.

## Provenance and peer review

It is not invited.
